# Metabolomics reveals mouse plasma metabolite responses to acute exercise and effects of disrupting AMPK-glycogen interactions

**DOI:** 10.3389/fmolb.2022.957549

**Published:** 2022-08-24

**Authors:** Mehdi R. Belhaj, Nathan G. Lawler, John A. Hawley, David I. Broadhurst, Nolan J. Hoffman, Stacey N. Reinke

**Affiliations:** ^1^ Exercise and Nutrition Research Program, Mary MacKillop Institute for Health Research, Australian Catholic University, Melbourne, VIC, Australia; ^2^ Centre for Integrative Metabolomics and Computational Biology, School of Science, Edith Cowan University, Joondalup, WA, Australia; ^3^ Australian National Phenome Centre and Centre for Computational and Systems Medicine, Health Futures Institute, Murdoch University, Perth, WA, Australia

**Keywords:** exercise metabolism, plasma metabolite, metabolomics, AMP-activated protein kinase, glycogen, amino acids, acylcarnitines, pantothenic acid

## Abstract

**Introduction:** The AMP-activated protein kinase (AMPK) is a master regulator of energy homeostasis that becomes activated by exercise and binds glycogen, an important energy store required to meet exercise-induced energy demands. Disruption of AMPK-glycogen interactions in mice reduces exercise capacity and impairs whole-body metabolism. However, the mechanisms underlying these phenotypic effects at rest and following exercise are unknown. Furthermore, the plasma metabolite responses to an acute exercise challenge in mice remain largely uncharacterized.

**Methods:** Plasma samples were collected from wild type (WT) and AMPK double knock-in (DKI) mice with disrupted AMPK-glycogen binding at rest and following 30-min submaximal treadmill running. An untargeted metabolomics approach was utilized to determine the breadth of plasma metabolite changes occurring in response to acute exercise and the effects of disrupting AMPK-glycogen binding.

**Results:** Relative to WT mice, DKI mice had reduced maximal running speed (*p* < 0.0001) concomitant with increased body mass (*p* < 0.01) and adiposity (*p* < 0.001). A total of 83 plasma metabolites were identified/annotated, with 17 metabolites significantly different (*p* < 0.05; FDR<0.1) in exercised (↑6; ↓11) versus rested mice, including amino acids, acylcarnitines and steroid hormones. Pantothenic acid was reduced in DKI mice versus WT. Distinct plasma metabolite profiles were observed between the rest and exercise conditions and between WT and DKI mice at rest, while metabolite profiles of both genotypes converged following exercise. These differences in metabolite profiles were primarily explained by exercise-associated increases in acylcarnitines and steroid hormones as well as decreases in amino acids and derivatives following exercise. DKI plasma showed greater decreases in amino acids following exercise versus WT.

**Conclusion:** This is the first study to map mouse plasma metabolomic changes following a bout of acute exercise in WT mice and the effects of disrupting AMPK-glycogen interactions in DKI mice. Untargeted metabolomics revealed alterations in metabolite profiles between rested and exercised mice in both genotypes, and between genotypes at rest. This study has uncovered known and previously unreported plasma metabolite responses to acute exercise in WT mice, as well as greater decreases in amino acids following exercise in DKI plasma. Reduced pantothenic acid levels may contribute to differences in fuel utilization in DKI mice.

## 1 Introduction

The benefits of exercise on metabolic health are widely appreciated. Exercise represents a substantial challenge to cellular and whole-body energy homeostasis, and a multitude of molecular metabolic responses including changes in circulating metabolites occur in response to exercise to help meet increased energy demands and preserve homeostasis (Hawley et al., 2014). One of the key regulators of energy homeostasis is the AMP-activated protein kinase (AMPK), a pivotal cellular energy sensing kinase that integrates intracellular and extracellular metabolic signals and regulates numerous downstream metabolic pathways to mobilize energy reserves and generate ATP, the energy currency of the cell (Garcia and Shaw, 2017). Through competitive binding of ATP, ADP and AMP, AMPK senses cellular energy status. Decreases in cellular energy levels (i.e., increased AMP/ATP ratio) in response to exercise lead to activation of AMPK. Once activated, AMPK switches on catabolic pathways including glycolysis and fat oxidation, while concurrently switching off anabolic processes including fat and protein synthesis (Garcia and Shaw, 2017). In addition, AMPK contains a carbohydrate binding module (CBM) within its β subunit (existing in two isoforms; β1 and β2) that allows AMPK to bind glycogen (Hudson et al., 2003; Polekhina et al., 2003; Polekhina et al., 2005), an important energy reserve of glucose utilized during exercise (van Loon et al., 2001), thereby contributing to its cellular energy sensing capacity (McBride et al., 2009).

**FIGURE 1:**
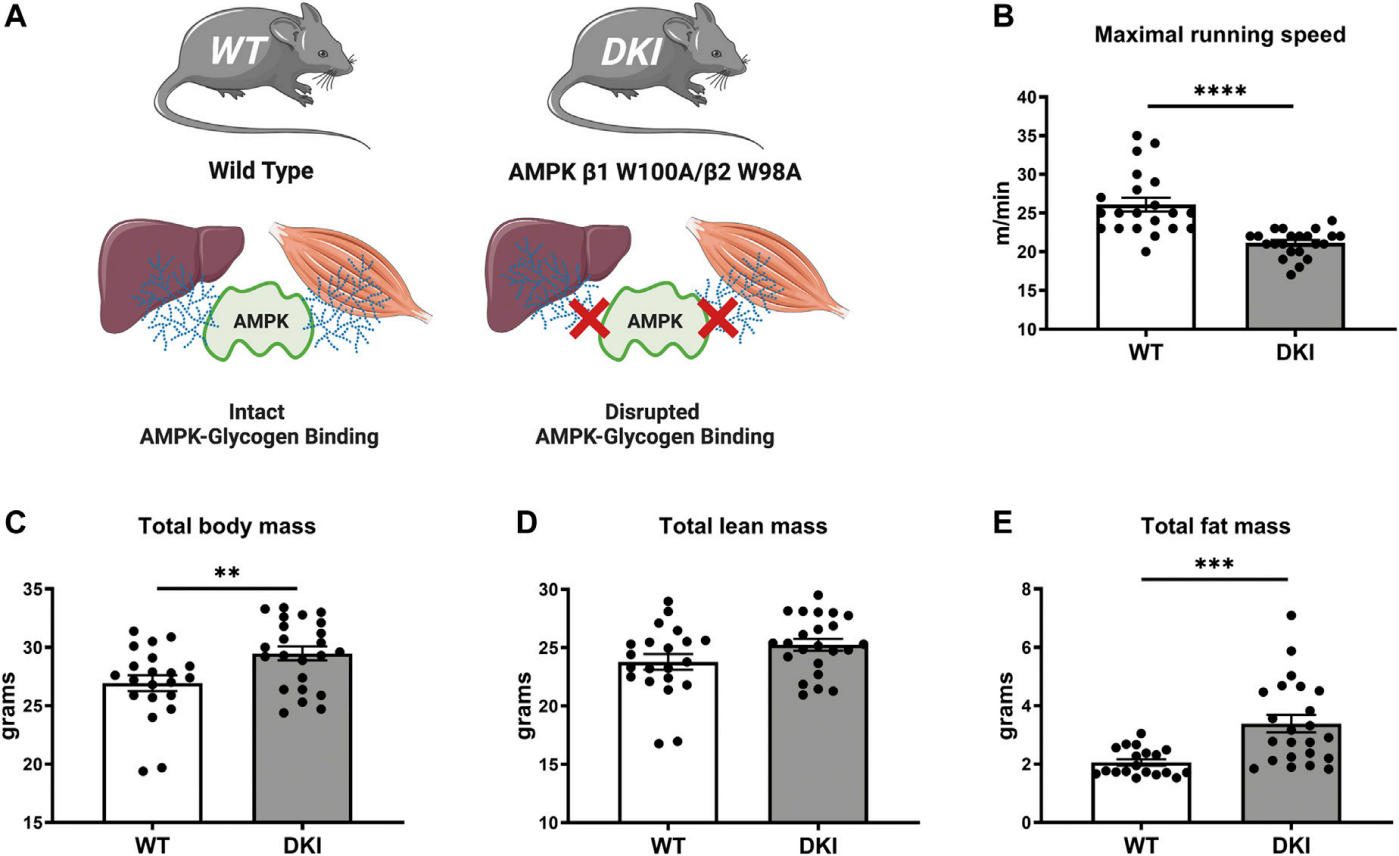
AMPK DKI mouse model and phenotypic effects of disrupting whole-body AMPK-glycogen interactions on maximal running speed and body composition. **(A)** Schematic of WT mice with intact AMPK-glycogen binding and AMPK DKI mice in which critical tryptophan residues within the AMPK β subunit isoforms that mediate glycogen binding (β1; predominantly expressed in mouse liver; and β2 predominantly expressed in mouse skeletal muscle) have been mutated to alanine (β1 W100A and β2 W98A, respectively), resulting in whole-body disruption of AMPK-glycogen binding; **(B)** Maximal running speed (m/min); **(C)** Total body mass (g); **(D)** Total lean mass (g); **(E)** Total fat mass (g). *: *p* < 0.05, **: *p* < 0.01, ****p < 0.0001; values are represented as mean ± SEM; *n* = 21–23 mice per group.

A growing body of evidence has uncovered interactive roles of AMPK and glycogen at the molecular, cellular and physiological levels (Janzen et al., 2018), including investigations using animal model systems with mutant forms of AMPK that would not be feasible to study in humans. Recent findings from our group demonstrated phenotypic effects of disrupting AMPK’s glycogen binding capacity *in vivo*, using AMPK β1 or β2 single knock-in (KI) mice in which amino acid residues in either isoform CBM that mediate glycogen binding were mutated (β1 W100A KI or β2 W98A KI) (Hoffman et al., 2020). These β1 W100A KI and β2 W98A KI mutations were associated with disrupted whole-body and tissue metabolism and impaired maximal running capacity in β2 W98A KI mice relative to wild type (WT) mice. Considering both AMPK β1 and β2 isoforms are expressed in the majority of mouse tissues and cell types including liver and skeletal muscle (Janzen et al., 2018), mice with KI mutations in both isoforms (AMPK β1 W100A/β2 W98A double knock-in; DKI; Figure 1A) were generated to investigate the phenotypic consequences of disrupting whole-body AMPK-glycogen interactions at rest and during exercise. DKI mice displayed impaired whole-body metabolism and changes in energy substrate utilization (i.e., reduced fat oxidation) associated with increased total body and fat mass and altered tissue glycogen dynamics (Janzen et al., 2021). Furthermore, these metabolic effects were associated with reduced maximal exercise capacity in DKI mice compared to WT (Janzen et al., 2022). Despite recent progress in the understanding of metabolic and physiological effects of disrupting AMPK-glycogen interactions, the downstream changes in metabolic pathways and molecular mechanisms underlying these effects remain largely unknown.

Discovery-based untargeted metabolomics can be used to investigate metabolic changes in response to acute exercise and in association with whole-body disruption of AMPK-glycogen binding. The metabolome reflects the cumulative changes that result from layers of biological regulation involving the genome, transcriptome and proteome, as well as their interactions with the environment and physiological stimuli such as exercise (Belhaj et al., 2021). Metabolomics therefore provides a snapshot of the metabolic reactions in “real-time” (Dunn et al., 2011). Using mass spectrometry-based untargeted metabolomic approaches, metabolites across a wide range of metabolic pathways can be simultaneously identified in blood and tissue samples, requiring only minimal sample volumes. Using untargeted metabolomics, we aimed to determine plasma metabolic responses to acute treadmill running and characterize the effects of disrupting AMPK-glycogen interactions on plasma metabolite profiles both at rest and following exercise. We sought to identify underlying molecular mechanisms that may contribute to the metabolic phenotypic effects and reduced exercise capacity observed in AMPK DKI mice.

## 2 Materials and methods

### 2.1 Animal models

All mouse procedures were performed under the approval of the St. Vincent’s Hospital (Melbourne, Australia) Animal Ethics Committee (approval numbers 025-15 and 011-19) in accordance with NHMRC requirements and conforming to the National Institutes of Health animal research guidelines (NIH Publications No. 8023, revised 1978) and Australian codes of animal research practice (8th Edition 2013). Mice were kept in pathogen-free microisolator cages (2–5 mice per cage) on standard 12:12-h dark-light cycles with controlled temperature (21°C), humidity and bedding. Mice had *ad libitum* access to water and standard chow diet (29% starch 20% protein and 6% fat, Barastoc, Ridley Agriproducts, Pakenham, Australia).

CRISPR/Cas9 gene editing was performed by the Mouse Engineering Garvan/ABR (MEGA) Facility to generate whole-body AMPK *Prkab1* ^W100A^ (β1 W100A) and *Prkab2* ^W98A^ (β2 W98A) single KI mice on a C57BL/6J background (Hoffman et al., 2020). Homozygous single KI mice were crossed to generate *Prkab1* ^W100A^/*Prkab2* ^W98A^ DKI mice. Breeding was performed using homozygous WT mice and homozygous carriers of the DKI mutations, to generate the homozygous WT and DKI mouse lines used in the present study. Due to limited age-matched mouse cohort availability and in order to maintain sufficient female mice for breeding, only male mice were used in the present study. Tail samples were collected for confirmatory genotyping performed by TransnetYX (Cordova, TN, United States). All experiments were performed in age-matched WT and DKI male mice within an age range of 12–16 weeks, and mice from each genotype were randomly assigned to the rested and exercised experimental groups.

### 2.2 Study design

#### 2.2.1 Treadmill acclimatization, body composition analysis and maximal running speed testing

Prior to treadmill acclimatization and subsequent maximal running speed testing, total body mass was determined, and body composition was analyzed by magnetic resonance imaging using the EchoMRI Body Composition Analysis 3-in-1 system (EchoMRI, Houston, TX, United States). Mice were then acclimatized to treadmill running (Exer 3/6, Columbus Instruments, Columbus, OH, United States) for four consecutive days at progressing speeds at a 0° incline. Manual prodding using a soft bristle brush was performed to encourage mice when necessary during both treadmill acclimatization and testing. On day 1, mice were placed on a stationary treadmill for 5 min. On days 2 and 3, mice ran for 5 min at 5 and 10 m/min, respectively. On day 4, mice ran for 15 min; including 5 min at 10, 12 and 15 m/min. The following day, mice underwent incremental maximal running capacity testing during which they ran for 2 min at 10 m/min, after which the speed was increased by 1 m/min every 2 min until exhaustion (i.e., when mice were unable to continue running despite repeated manual prodding). Both WT (*n* = 21) and DKI (*n* = 23) mice were then randomly assigned to either the exercised or rested control group, with a total of four experimental groups: WT rested (WT-Rest; *n* = 10), WT exercised (WT-Ex; *n* = 11), DKI rested (DKI-Rest; *n* = 13) and DKI exercised (DKI-Ex; *n* = 10).

#### 2.2.2 Acute treadmill exercise bout and blood sample collection

Following two to 3 days of recovery after maximal running capacity testing, mice randomly assigned to the exercise group completed a single bout of running for 30 min at 70% of individual maximal speed at 0° treadmill incline, while rested control mice remained in their home cage. This 70% of individual maximal running speed was used due to the mix of carbohydrate and fat energy sources expected to be utilized at this speed (Torgan et al., 1990; McClelland, 2004). Upon completion of the rest period or acute exercise bout, rested and exercised mice were immediately placed in a CO_2_ chamber for ∼10 s prior to a cardiac puncture to allow sufficient blood volume collection. Blood collections were performed in the fed state between 0800 and 1000 h in both the exercised and rested groups, corresponding to the beginning of the light phase. Blood was collected *via* cardiac puncture using a 23G needle attached to a 1 ml syringe, immediately transferred to 1.3 ml lithium-heparin coated tubes (Sarstedt, Nümbrecht, Germany), inverted 10 times and placed on ice. Tubes were then centrifuged at 2000 × *g* for 10 min at 4°C, with total time on ice consistent for all collected samples. The resulting plasma supernatant was then divided into 300 μl aliquots and stored at −80°C until analysis.

### 2.3 Metabolomics sample preparation

Metabolomic analysis of mouse plasma samples was performed at the Centre for Integrative Metabolomics and Computational Biology, Edith Cowan University (Joondalup, Western Australia, Australia) according to previously established data acquisition, processing and analysis workflows (Manaf et al., 2018). Plasma samples were thawed on ice and each sample was separated into two 30 μl aliquots. A pooled quality control (QC) sample was prepared by mixing 50 μl of remaining plasma from each sample; the pooled sample was subsequently divided into 30 μl aliquots. All prepared aliquots were then frozen and stored at −80°C until analysis.

On the day of mass spectrometry-based metabolomic data acquisition, plasma aliquots were thawed on ice, and protein precipitation was performed prior to running two liquid chromatography-based separation modes, as detailed below. For hydrophilic interaction liquid chromatography (HILIC), protein precipitation was performed by adding 90 μl of ice-cold liquid chromatography-mass spectrometry (LC-MS) grade acetonitrile [3:1 (v/v)] containing isotopically labelled standards of known concentration [1 ppm each: valine-d8, tryptophan-d9, taurodeoxycholic acid-d4, leucine-13C1 and sphingosine-d9 (d18:1)] to plasma samples and pooled QC aliquots. For reversed-phase liquid chromatography (RPLC), protein precipitation was performed by adding 90 μl of ice-cold LC-MS grade methanol to each 30 μl aliquot, with identical internal standards. Samples were then mixed at 1,400 revolutions per min (rpm) for 2 min at 4°C and centrifuged (Heraeus Megafuge 8R, Thermo Fisher Scientific. AUS) for 20 min (1800 × *g*) at 4°C. 30 μl from each of the resulting supernatants were transferred into LC-MS vials and placed in an autosampler tray at 6°C. Prior to the run, the sample injection sequence was block randomized by group (WT-Rest, WT-Ex, DKI-Rest, DKI-Ex). LC-MS system suitability was checked using the quality assurance methods described by Broadhurst et al. (2018) and eight pooled QC samples were injected at the beginning of the sequence to condition the column. Pooled QC samples were then injected following each block of eight experimental samples (i.e., every ninth injection) which were used for assessment of analytical precision as reported in (Broadhurst et al., 2018).

### 2.4 Data acquisition: Liquid chromatography–high resolution mass spectrometry

Samples were analyzed using the Dionex UltiMate 3000™ platform which comprises an ultra-high performance liquid chromatography pump coupled to a heated electrospray Q Exactive Focus Orbitrap mass spectrometer (Thermo Scientific, San Jose, CA, United States). Data were acquired in full scan mode at a mass resolution of 70,000 [Full Width at Half Maximum (FWHM) at mass to charge ratio (*m/z*) of 200], over a scan range of *m/*z 70–1000. Tandem mass spectrometry (MS/MS) was performed on all QC samples using data-dependent acquisition (DDA) in “discovery” mode using the following settings: resolution = 17,500; isolation width = 3.0 m/z; stepped collision energy = 15, 30 eV. Source and ion transfer conditions were as follows: sheath gas = 35 (arbitrary units), auxiliary gas = 10 (arbitrary units), source heater 350°C, capillary temperature = 350°C, ion spray voltage = 2.5 kV (negative ion mode) and 3.0 kV (positive ion mode). The automatic gain control target was set at 3 × 10^–6^.

Two chromatography-based separation methods were applied to each sample. Polar metabolites were separated by HILIC chromatography in positive mode (HILIC POS), using an ACQUITY UPLC BEH Amide column (2.1 × 100 mm, 1.7 μm; Waters, Milford, MA, United States) with a flow rate of 400 μl/min and the following mobile phases: mobile phase A (10 mM ammonium formate in water +50 mM formic acid) and mobile phase B (10 mM ammonium formate in 90% Acetonitrile +50 mM formic acid). A 15-min elution gradient was applied as follows: 0 min; 100% B; 1 min 1% B; 8–9 min 60% B; 10 min 100% B through to 15 min. A 6 μl sample injection volume was applied. Non-polar metabolites were separated by RPLC on a Hypersil GOLD column (2.1 × 100 mm, 1.9 μm; Thermo Fisher Scientific, Runcorn, UK), with a flow rate 300 μl/min using the following mobile phases: mobile phase A (water +0.1% formic acid) and mobile phase B (Acetonitrile +0.1% formic acid). A 15-min elution gradient was applied as follows: 0 min 1% B; 1 min 1% B; 2 min 50% B; 9–10 min 99% B; 10.5 min 1% B through to 15 min. A 6 μl sample injection was applied, and samples were collected in positive and negative mode. Xcalibur software (Thermo Fisher Scientific v4.3) was used for data acquisition. Before analysis, commercially purchased calibrant solutions (negative and positive ion calibration solutions) from Thermo Fisher Scientific (San Jose, CA, United States) were used to externally calibrate the Orbitrap mass spectrometer.

### 2.5 Data processing

Following data acquisition, spectral processing was performed (i.e., HILIC POS and C18 POS and C18 NEG) in Compound Discoverer™ 3.1 (Thermo Scientific, San Jose, CA, United States) using an untargeted metabolomics workflow. Compound Discoverer™ version 3.1 was utilized for total ion chromatograms alignment along retention times (RT) based on an adaptive curve, with a 5-ppm mass tolerance and a maximum shift of 0.5 min. Detected features with a signal-to-noise ratio >5 and an intensity ≥1,000,000 in each dataset were merged into compounds according to ion adducts. Ions detected within the blanks were subtracted from the samples using the “mark background compounds” node.

Metabolite identification/annotation was also performed in Compound Discoverer™ in accordance with the Metabolomics Standard Initiative (MSI) levels (Sumner et al., 2007). Details on identifications/annotations can be found in Supplementary Table S2. Quality control-regularized spline correction (QC-RSC) was used to correct analytical signal drift (Kirwan et al., 2013). Following standard protocols (Broadhurst et al., 2018), metabolites with RSD_QC_ > 20% or a Dispersion-ratio (D-ratio) > 30% were removed from further statistical analyses based on their failure to meet acceptable measurement precision. Eight hemolyzed plasma samples were removed from the full sample set to prevent potential confounding effects of sample hemolysis on the metabolomic analysis.

### 2.6 Statistical analyses

For statistical analyses of mouse body mass, body composition and maximal running speed, *p-*values were calculated using unpaired Student’s *t-*tests (WT versus DKI) and Welch’s correction was applied when applicable, with *p* < 0.05 considered statistically significant. Outlier identification was performed using the Grubb’s test or the ROUT method. Statistical tests for these measures were performed using GraphPad Prism software version 8.4.3 (GraphPad Software, San Diego, CA).

For statistical analyses of the metabolomics dataset, two-way ANOVA was performed to test the null hypothesis (i.e., no differences between rested/exercised conditions and/or WT/DKI genotypes) for each individual identified/annotated metabolite. Estimation of the false discovery rate (FDR) due to multiple comparisons was performed using the Storey FDR method (Storey, 2002), and *p*-values < 0.05 with a FDR <0.1 were considered statistically significant.

To assess the structured similarities between metabolite concentrations across the complete data set, hierarchical cluster analysis (HCA) was performed using Pearson’s correlation coefficient as the similarity measure, and Ward’s linkage method (Ward and Hook, 1963). The lowest linkages (i.e., closest metabolite clusters) within the HCA dendrogram indicate metabolites that display the most similar responses. The resulting visualization labeled by metabolite significance, indicates metabolites of particular interest displaying similar (correlated) changes.

To model and visualize the multivariate discrimination between the four experimental groups, all metabolites were combined into a single data matrix and applied to principal component–canonical variates analysis (PC-CVA) (Krzanowski, 1988). PC-CVA uses the (multivariate) covariance between metabolite concentration and outcome classification to test the statistical significance of the mean differences. Multivariate data is projected into a two-dimensional subspace (canonical variate axes) reflecting the combination of metabolites that best discriminate the labeled groups. Results are visualized *via*: 1) a scores plot, with 95% confidence intervals (CI) of the mean from each group indicating significant differences, and 2) a coefficients plot indicating the contribution importance of each metabolite to each canonical variate. Overfitting was avoided by using 5-fold cross validation to choose the optimal number of principal components to apply to the CVA model. 95% CI for the model coefficients were calculated using bootstrap resampling (Hastie et al., 2009). Prior to all statistical analyses, metabolite profiles were log_10_ transformed and scaled to unit variance (autoscaled), allowing each metabolite to be objectively compared with no bias due to large differences in metabolite concentration. Statistical analyses of the metabolomic dataset were performed using MATLAB® package version R2021b (MathWorks, Natick, MA).

## 3 Results

### 3.1 AMP-activated protein kinase double knock-in mice exhibit increased total body mass, fat mass and reduced maximal running speed

Compared to age-matched WT mice (*n* = 21), DKI mice (*n* = 23) with whole-body disruption of AMPK-glycogen binding (Figure 1A) displayed ∼20% reduction in maximal running speed (*p* < 0.0001, Figure 1B). DKI mice had increased total body mass (∼10%, *p* < 0.01, Figure 1C) associated with increased whole-body fat mass (∼65%, *p* < 0.001) but no differences in lean mass (Figures 1D,E) relative to WT mice.

### 3.2 Metabolomics reveals plasma metabolites affected by acute treadmill exercise and/or AMP-activated protein kinase double knock-in genotype

A total of 83 metabolites were identified/annotated following data processing and cleaning steps and according to the standard reporting guidelines from the Metabolomics Society Initiative (Sumner et al., 2007). Two-way ANOVA revealed 17 metabolites were significantly different (*p* < 0.05; FDR <0.1) between conditions (rested versus exercised) and only one metabolite significantly different between genotypes (WT versus DKI). All identified/annotated metabolites, in addition to their molecular characteristics and respective statistical relationships are listed in Table 1. Furthermore, Table 2 reports metabolite change directionality, median fold-change (FC) and 95% confidence intervals (CI) of these 17 metabolites that significantly changed between experimental groups along with their associated pathways. All metabolite information and statistics are reported in Supplementary Tables S1–S3.

**TABLE 1:** Summary of the metabolites identified/annotated from WT and DKI mouse plasma. A total of 83 plasma metabolites were identified/annotated, including 17 metabolites significantly changed by condition (i.e., exercise versus rest) and one metabolite significantly changed by genotype (i.e., DKI versus WT) based on univariate analysis. Statistically significant values (*p* < 0.05 and/or FDR<0.1) appear in bold text. C, condition; G, genotype; G × C, interaction between condition and genotype; FDR. false discovery rate; MSI, Metabolomics Standards Initiative; RSD_QC_, relative standard deviation in quality control samples; RT, retention time.

Column & mode	Metabolite	Molecular formula	Molecular weight	RT (min)	RSD_QC_	*p*-value G	FDR G	*p*-value C	FDRC	*p*-value GxC	FDR GxC	MSI ID	Cluster
C18POS	alpha-Linolenic acid	C18 H30 O2	278.2241	8.27	10.918	0.911	0.454	0.657	0.658	0.429	0.519	1	A
C18NEG	C18:1 fatty acid	C18 H34 O2	282.2551	9.67	6.993	0.230	0.263	0.355	0.440	0.548	0.529	2	A
C18POS	Arachidonic acid	C20 H32 O2	304.2401	8.72	9.285	0.321	0.295	0.791	0.691	0.294	0.542	2	A
C18POS	2-Arachidonoyl glycerol	C23 H38 O4	378.2764	8.17	13.736	0.250	0.260	0.915	0.685	0.093	0.444	2	A
C18POS	Monoolein	C21 H40 O4	356.2924	8.93	10.887	0.237	0.258	0.985	0.685	0.074	0.377	2	A
HILICPOS	Lyso-PAF C16	C24 H52 N O6 P	481.3527	2.34	13.091	**0.006**	0.145	0.323	0.432	0.182	0.484	1	A
C18NEG	Bile acid (C26H45NO7S)	C26 H45 N O7 S	515.2906	3.63	3.804	**0.014**	0.222	0.846	0.702	0.356	0.483	3	A
C18NEG	Stercobilin	C33 H46 N4 O6	594.3399	4.75	8.386	0.259	0.264	0.858	0.695	0.983	0.661	2	A
C18NEG	Lithocholyltaurine	C26 H45 N O5 S	483.3010	4.61	4.708	0.185	0.271	0.952	0.675	0.381	0.497	1	A
C18POS	Taurochenodesoxycholic acid	C26 H45 N O6 S	499.2965	4.13	2.108	0.052	0.220	0.992	0.683	0.251	0.538	1	A
C18NEG	Chenodeoxycholic acid glycine conjugate	C26 H43 N O5	449.3133	4.29	4.633	0.091	0.225	0.880	0.681	0.201	0.496	1	A
C18POS	Glycocholic acid	C26 H43 N O6	465.3088	4.25	6.866	0.081	0.211	0.924	0.684	0.175	0.528	1	A
C18NEG	Glycoursodeoxycholic acid	C26 H43 N O5	449.3134	4.92	2.424	0.080	0.221	0.773	0.692	**0.048**	0.318	2	A
C18NEG	Riboflavin	C17 H20 N4 O6	376.1374	3.33	5.997	0.151	0.294	0.940	0.674	0.102	0.399	2	A
C18NEG	Oxidized glutathione	C20 H32 N6 O12 S2	612.1506	1.62	7.792	0.339	0.299	0.863	0.691	0.172	0.545	1	A
C18POS	Reduced glutathione	C10 H17 N3 O6 S	307.0836	1.30	15.021	**0.019**	0.177	0.405	0.493	0.335	0.519	1	A
C18POS	Glycylproline	C7 H12 N2 O3	172.0847	1.29	8.734	**0.015**	0.178	0.869	0.688	0.318	0.516	1	B
HILICPOS	Propionylcarnitine	C10 H19 N O4	217.1312	3.25	8.578	**0.036**	0.212	0.105	0.238	0.849	0.657	1	B
C18NEG	Bile acid (C24H40O5)	C24 H40 O5	408.2866	4.12	3.099	0.053	0.208	0.913	0.691	0.696	0.586	3	B
C18NEG	Chenodeoxycholic acid	C24 H40 O4	392.2919	4.89	7.111	0.342	0.297	0.133	0.266	0.152	0.506	1	B
C18POS	Nutriacholic acid	C24 H38 O4	390.2767	5.09	5.142	0.285	0.272	0.133	0.259	0.406	0.501	1	B
C18NEG	Deoxycholic acid	C24 H40 O4	392.2921	5.75	4.756	0.201	0.262	0.252	0.381	0.096	0.426	2	B
HILICPOS	Hypotaurine	C2 H7 N O2 S	109.0199	5.88	12.036	0.523	0.340	0.876	0.686	0.808	0.632	1	B
C18NEG	Thymidine	C10 H14 N2 O5	242.0897	2.98	5.315	0.991	0.473	0.480	0.554	0.245	0.563	2	B
HILICPOS	Acetylcarnitine	C9 H17 N O4	203.1154	4.11	11.964	0.620	0.368	0.245	0.380	0.348	0.492	1	B
HILICPOS	Bilirubin	C33 H36 N4 O6	584.2620	0.91	6.493	0.895	0.451	0.794	0.684	0.073	0.404	2	B
HILICPOS	Carnitine	C7 H15 N O3	161.1049	4.11	11.349	0.384	0.321	0.329	0.430	0.127	0.470	1	C
HILICPOS	Melatonin	C13 H16 N2 O2	232.1209	0.82	10.277	**0.041**	0.193	0.256	0.379	0.301	0.527	1	C
C18POS	Cytosine	C4 H5 N3 O	111.0435	1.30	4.534	0.454	0.322	**0.024**	**0.088**	0.963	0.661	2	C
C18POS	Cytidine	C9 H13 N3 O5	243.0853	1.29	6.709	0.515	0.345	0.983	0.690	0.925	0.655	1	C
C18POS	Kynurenic acid	C10 H7 N O3	189.0423	3.24	2.819	0.867	0.442	0.198	0.338	0.918	0.657	1	C
C18POS	Spermidine	C7 H19 N3	145.1579	0.77	7.732	0.530	0.335	0.266	0.377	0.733	0.609	1	C
HILICPOS	Stearoylethanolamide	C20 H41 N O2	327.3132	0.66	7.501	0.281	0.274	0.126	0.268	0.341	0.505	1	C
HILICPOS	Dipalmitoylphosphatidylcholine	C40 H80 N O8 P	733.5612	0.98	6.336	0.635	0.363	0.057	0.170	0.796	0.631	2	C
C18NEG	N-Acetyltyrosine	C11 H13 N O4	223.0835	3.22	8.121	0.430	0.325	0.105	0.231	0.691	0.605	1	C
C18POS	N-Acetyltryptophan	C13 H14 N2 O3	246.1002	3.66	4.804	0.552	0.344	**0.010**	**0.044**	0.547	0.536	1	C
C18POS	Progesterone	C21 H30 O2	314.2238	6.38	6.020	0.165	0.298	**1.09E-09**	**3.72E-08**	**0.030**	0.334	1	C
C18POS	Corticosterone	C21 H30 O4	346.2142	4.36	5.214	0.414	0.329	**5.11E-10**	**3.48E-08**	0.889	0.665	2	C
HILICPOS	Palmitoylcarnitine	C23 H45 N O4	399.3339	0.97	6.812	0.944	0.461	**0.001**	**0.005**	0.895	0.662	1	C
C18NEG	16-Hydroxyhexadecanoic acid	C16 H32 O3	272.2347	8.44	6.147	0.527	0.338	0.097	0.228	0.256	0.533	2	C
HILICPOS	Hexanoylcarnitine	C13 H25 N O4	259.1777	1.72	13.234	0.738	0.402	**0.024**	**0.092**	0.495	0.540	2	C
C18NEG	Hexadecanedioic acid	C16 H30 O4	286.2140	6.12	12.155	0.210	0.266	0.750	0.681	0.953	0.660	2	C
C18POS	Histamine	C5 H9 N3	111.0799	0.80	2.585	0.458	0.320	0.677	0.659	0.866	0.654	1	C
HILICPOS	Methylimidazoleacetic acid	C6 H8 N2 O2	140.0585	5.26	10.576	0.495	0.336	0.304	0.423	0.748	0.607	1	C
HILICPOS	Prolylleucine/Leucylproline	C11 H20 N2 O3	228.1468	5.10	13.965	0.357	0.304	0.503	0.571	0.977	0.663	2	D
HILICPOS	Creatine	C4 H9 N3 O2	131.0694	5.84	6.064	0.273	0.272	0.197	0.343	0.396	0.507	1	D
C18POS	Methylthioadenosine	C11 H15 N5 O3 S	297.0894	3.19	2.489	0.169	0.282	0.339	0.428	0.607	0.561	1	D
C18NEG	Fructose 6-phosphate	C6 H13 O9 P	260.0290	0.78	6.551	0.113	0.252	0.677	0.668	0.504	0.524	1	D
C18POS	Serotonin	C10 H12 N2 O	176.0949	2.92	4.198	0.141	0.287	0.713	0.674	0.642	0.586	1	D
C18NEG	12-HETE	C20 H32 O3	320.2346	6.83	5.510	0.600	0.365	0.725	0.676	0.589	0.552	1	D
C18POS	Eicosapentaenoic acid	C20 H30 O2	302.2243	6.83	6.254	0.438	0.326	0.683	0.655	0.692	0.598	2	D
HILICPOS	Hypoxanthine	C5 H4 N4 O	136.0385	3.64	10.615	0.441	0.323	0.266	0.385	0.462	0.549	1	D
HILICPOS	Adenosine	C10 H13 N5 O4	267.0961	2.25	17.382	0.577	0.355	0.192	0.345	0.574	0.548	1	D
C18NEG	Guanosine monophosphate	C10 H14 N5 O8 P	363.0571	1.27	6.942	0.170	0.275	0.507	0.557	0.694	0.592	1	D
C18POS	Adenosine monophosphate	C10 H14 N5 O7 P	347.0631	0.90	1.401	0.691	0.385	0.803	0.684	0.682	0.614	1	D
C18POS	Oleamide	C18 H35 N O	281.2714	9.16	4.790	0.236	0.263	0.433	0.508	0.738	0.606	1	E
C18POS	Hexadecanamide	C16 H33 N O	255.2559	9.23	8.763	0.167	0.290	0.556	0.591	0.484	0.555	2	E
HILICPOS	Nicotinamide 1-oxide	C6 H6 N2 O2	138.0426	2.04	4.087	0.797	0.429	0.777	0.688	0.486	0.539	2	E
C18POS	Niacinamide	C6 H6 N2 O	122.0481	1.01	11.716	0.420	0.328	0.892	0.682	0.314	0.536	1	E
HILICPOS	4-Guanidinobutyric acid	C5 H11 N3 O2	145.0851	4.47	15.185	0.854	0.444	0.072	0.195	0.175	0.506	2	E
C18NEG	Indoxyl sulfate	C8 H7 N O4 S	213.0087	3.27	4.325	0.404	0.326	0.543	0.587	0.860	0.658	1	E
C18POS	Phenylacetylglycine	C10 H11 N O3	193.0740	3.52	5.660	0.671	0.379	0.228	0.362	0.909	0.665	1	E
C18POS	2-Aminooctanoic acid	C8 H17 N O2	159.1260	3.52	3.860	0.197	0.272	0.224	0.364	0.317	0.527	1	E
C18POS	Tryptophan	C11 H12 N2 O2	204.0898	3.23	4.157	0.216	0.259	0.339	0.435	0.401	0.503	1	E
C18POS	Indole	C8 H7 N	117.0580	3.23	5.123	0.170	0.266	0.412	0.492	0.520	0.533	1	E
C18POS	Indoleacetic acid	C10 H9 N O2	175.0633	3.84	6.904	0.404	0.332	0.824	0.693	0.273	0.534	1	E
C18POS	3-Indolepropionic acid	C11 H11 N O2	189.0789	4.05	8.222	0.297	0.278	**0.030**	0.101	0.917	0.663	1	E
C18NEG	C18:2 fatty acid	C18 H32 O2	280.2398	6.34	3.507	0.424	0.325	**0.050**	0.154	0.296	0.533	3	E
C18POS	Sphingosine 1-phosphate	C18 H38 N O5 P	379.2484	5.78	2.156	0.073	0.245	**0.005**	**0.028**	0.690	0.612	1	E
C18POS	Pantothenic acid	C9 H17 N O5	219.1105	3.06	3.550	**0.001**	**0.070**	**5.50E-04**	**0.003**	0.287	0.545	1	F
C18POS	Methionine	C5 H11 N O2 S	149.0510	1.31	5.523	0.939	0.463	**3.13E-04**	**0.002**	**0.026**	0.347	1	F
C18POS	Ornithine	C5 H12 N2 O2	132.0899	0.80	1.918	0.223	0.261	**1.06E-04**	**0.001**	0.099	0.410	1	F
C18POS	Histidine	C6 H9 N3 O2	155.0695	0.81	2.298	**0.029**	0.227	**1.48E-05**	**2.02E-04**	0.266	0.537	1	F
C18POS	Phenylalanine	C9 H11 N O2	165.0789	2.96	5.817	0.726	0.400	0.084	0.205	0.327	0.519	1	F
HILICPOS	Tyrosine	C9 H11 N O3	181.0737	5.34	6.177	0.053	0.192	0.180	0.331	**0.039**	0.368	1	F
HILICPOS	Proline	C5 H9 N O2	115.0634	5.33	10.072	0.094	0.219	**0.024**	**0.096**	**0.009**	0.150	2	F
C18POS	Arginine	C6 H14 N4 O2	174.1116	0.84	3.661	0.631	0.369	**0.007**	**0.032**	**0.003**	0.198	1	F
HILICPOS	Isoleucine/Leucine	C6 H13 N O2	131.0946	4.78	6.852	0.604	0.363	**1.08E-04**	**9.19E-04**	**0.041**	0.344	1	F
C18POS	Pipecolic acid	C6 H11 N O2	129.0791	0.80	6.023	0.076	0.237	**2.70E-06**	**6.12E-05**	**0.005**	0.173	1	F
C18POS	Lysine	C6 H14 N2 O2	146.1055	0.80	3.389	0.121	0.258	**3.52E-06**	**5.99E-05**	**0.007**	0.160	1	F
C18POS	Glycerophospho-N-palmitoyl ethanolamine (GP-NPEA)	C21 H44 N O7 P	453.2853	6.82	2.864	0.244	0.260	**1.96E-04**	**0.001**	0.051	0.306	2	F
C18POS	Threonine/Homoserine	C4 H9 N O3	119.0583	0.88	2.437	0.480	0.330	0.070	0.199	**0.043**	0.314	1	F
C18POS	Testosterone	C19 H28 O2	288.2085	5.04	9.679	0.211	0.260	0.130	0.268	0.525	0.522	1	F

**TABLE 2:** Summary of the 17 metabolites significantly changed by exercise and/or genotype. A total of six metabolites significantly increased and 11 metabolites significantly decreased following exercise, at the univariate level. Increased metabolites primarily consisted of acylcarnitines and steroid hormones, while decreased metabolites predominantly consisted of amino acids and derivatives. Only pantothenic acid was significantly decreased in DKI versus WT mice at the univariate level. CI: confidence interval; FC: fold-change; **↑**: increase; **↓**: decrease.

	Metabolite	Pathway	Median FC and 95% CI (WT)	Median FC and 95% CI (DKI)	Cluster
Exercise vs. Rest	↑Cytosine	Pyrimidines	1.11 (0.78–1.62)	1.07 (0.95–1.43)	C
	↑N-Acetyltryptophan	Amino acids and derivatives	1.25 (0.83–2.07)	1.60 (1.01–2.78)	C
	↑Progesterone	Steroid hormones	1.98 (1.13–2.39)	2.48 (1.87–3.30)	C
	↑Corticosterone	Steroid hormones	4.74 (2.99–15.01)	5.79 (3.92–23.90)	C
	↑Palmitoylcarnitine	Acylcarnitines	1.37 (0.95–1.94)	1.73 (1.31–2.94)	C
	↑Hexanoylcarnitine	Acylcarnitines	1.31 (0.83–2.08)	1.48 (1.01–2.01)	C
	↓Sphingosine 1-phosphate	Sphingolipids	0.86 (0.74–0.97)	0.90 (0.74–1.00)	E
	↓Pantothenic acid	Vitamins and co-factors	0.78 (0.54–1.03)	0.79 (0.72–0.88)	F
	↓Methionine	Amino acids and derivatives	0.90 (0.73–1.07)	0.73 (0.68–0.90)	F
	↓Ornithine	Amino acids and derivatives	0.75 (0.57–1.05)	0.51 (0.38–0.67)	F
	↓Histidine	Amino acids and derivatives	0.67 (0.50–1.00)	0.67 (0.49–0.82)	F
	↓Proline	Amino acids and derivatives	1.25 (0.90–1.56)	0.60 (0.48–0.83)	F
	↓Arginine	Amino acids and derivatives	1.02 (0.86–1.40)	0.71 (0.53–0.86)	F
	↓Isoleucine/leucine	Amino acids and derivatives	0.89 (0.69–1.04)	0.69 (0.54–0.80)	F
	↓Pipecolic acid	Amino acids and derivatives	0.83 (0.67–1.02)	0.57 (0.48–0.78)	F
	↓Lysine	Amino acids and derivatives	0.78 (0.68–0.93)	0.59 (0.52–0.75)	F
	↓GP-NPEA	Glycerophospholipids	0.96 (0.81–1.19)	0.77 (0.67–0.91)	F
	**Metabolite**	**Pathway**	**Median fold change and 95% CI (rest)**	**Median fold change and 95% CI (exercise)**	**Cluster**
DKI vs. WT	↓Pantothenic acid	Vitamins and co-factors	0.82 (0.67–0.97)	0.83 (0.61–0.98)	F

#### 3.2.1 Clustering of correlated metabolites

**FIGURE 2:**
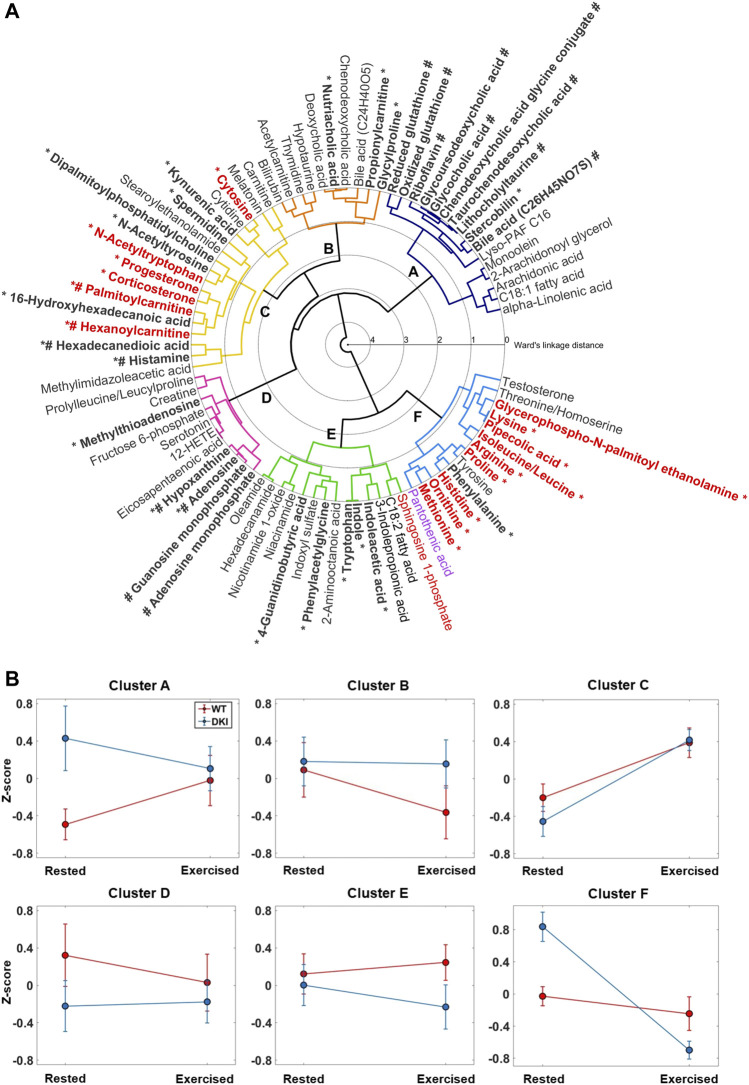
Hierarchical Cluster Analysis (HCA) dendrogram of identified/annotated metabolites. **(A)** Agglomerative clustering of individual metabolites based on pairwise correlation is shown. The lowest linkages within the HCA dendrogram indicate metabolites that display similar relative responses between the experimental groups. Six clusters were observed. Metabolite labels are colored to reflect the results of the two-way ANOVA after filtering using a false discovery rate (FDR) of 0.1 (red = significant effect of condition only; magenta = significant effect of genotype and condition; black = no significance or FDR >0.1). *: Metabolites that significantly (*p* < 0.05) contributed to the model along canonical variate 1 (CV1, Figure 3A); #: Metabolites that significantly (*p* < 0.05) contributed to the model along CV2. **(B)** Z-scores plot of the mean responses for each metabolite cluster. After conversion of individual metabolite log_10_ responses to a z-score, the average response of each cluster was calculated and presented here as a group error bar plot. Error bars indicate the standard error for each group mean (Red = WT; Blue = DKI). Following two-way ANOVA, only Clusters C and F showed significant differences in the averaged group metabolite response. Cluster C showed a significant effect of exercise for both genotypes (*p* = 2 × 10^−5^), and Cluster F showed a significant interaction between genotype and exercise (*p* = 4 × 10^−4^) such that the WT metabolite levels remained constant and the DKI metabolite levels significantly decreased in response to exercise.

Hierarchical clustering of the 83 metabolites, based on pair-wise Pearson’s correlation similarity, resulted in six clusters reflecting different observed interaction between genotype and exercise. The results are displayed as a circular dendrogram (Figure 2A). The averaged z-score metabolite responses for each cluster are presented in Figure 2B. The average response for metabolites in Cluster A (16 metabolites, including fatty and bile acids) was non-significant but showed an interaction between genotype and exercise such that during rest the DKI metabolite level was greater than the WT level, but those levels converged during exercise. Cluster B (10 metabolites, including bile acids, acetylcarnitine and hypotaurine), again non-significant, showed the opposite trend (converged at rest and lower WT following exercise). Cluster C (18 metabolites, including steroid hormones and acylcarnitines), showed a significant increase in metabolite levels associated with exercise in both genotypes (*p* = 2 × 10^−5^). Cluster D (11 metabolites, including nucleotides and derivatives, lipid species and creatine) and Cluster E (14 metabolites, including lipid species, tryptophan and derivatives) showed similar non-significant trends as Cluster A and B but with the WT metabolite levels greater than DKI. Cluster F (14 metabolites, including amino acids and derivatives, pantothenic acid and testosterone) showed a significant interaction between genotype and exercise (*p* = 4 × 10^−4^) such that the WT metabolite levels remain constant and the DKI metabolite levels significantly decrease in association with exercise. Clusters C and F drove the metabolite profile response to exercise intervention; *p*-values of all metabolite clusters are presented in Supplementary Table S3.

#### 3.2.2 Principal component-canonical variate analysis

**FIGURE 3:**
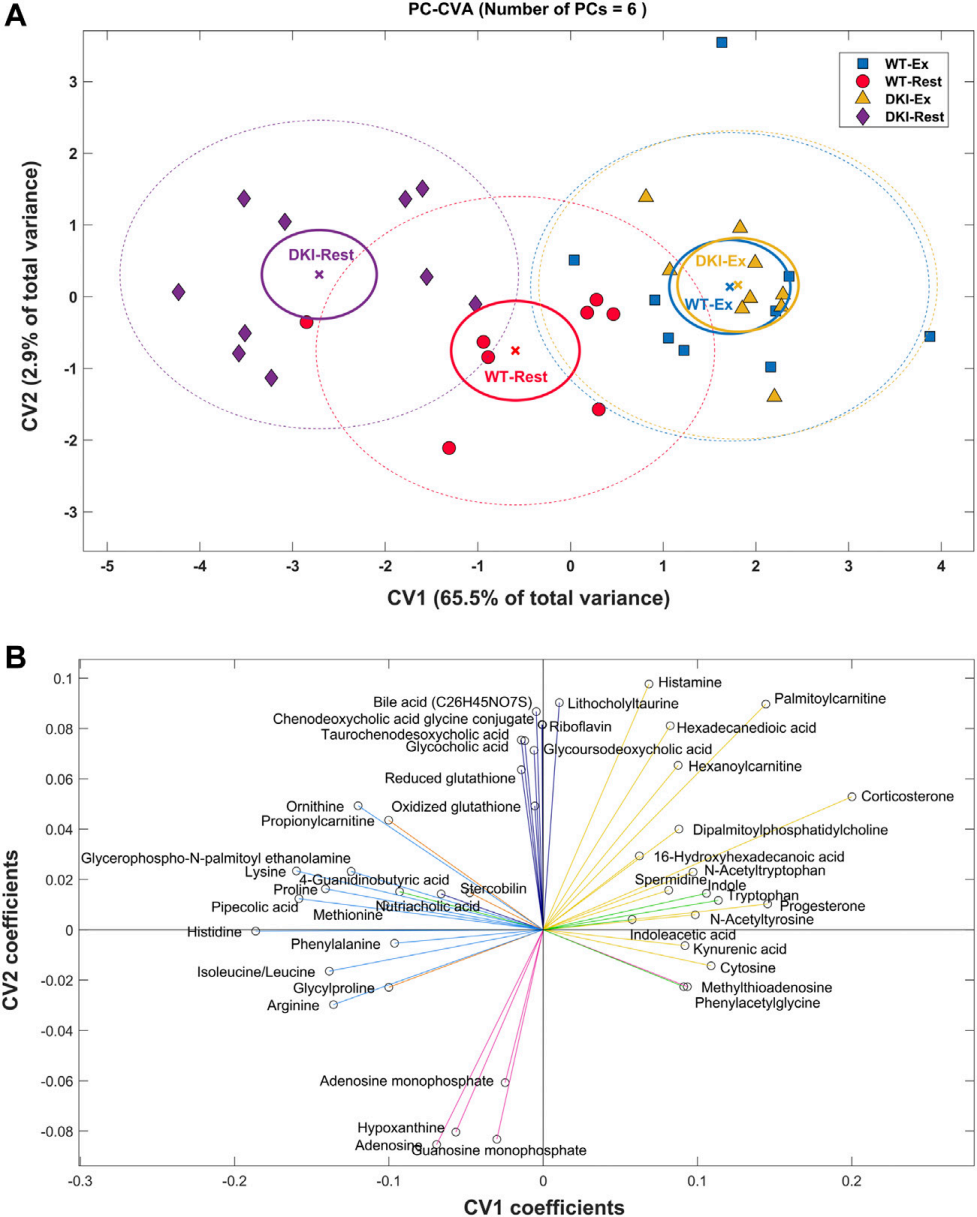
Principal Component-Canonical Variates Analysis (PC-CVA) showing system-wide metabolite profile differences between genotype at rest and after exercise. **(A)** Scores plot of Canonical Variate 1 (CV1) vs. Canonical Variate 2 (CV2). Each point (circle, square or triangle) represents a single sample [WT-Rest (*n* = 8), WT-Ex (*n* = 9), DKI-Rest (*n* = 10), DKI-Ex (*n* = 9)]. The mean (x) of each group is surrounded by a 95% confidence interval of the mean (full-line circles) and 95% confidence interval of membership in each sample group (dashed-line circles). Sample group means are considered significantly different when the 95% confidence interval of the means do not overlap. **(B)** The loading plot shows the influence (model coefficient value) of each metabolite that significantly (*p* < 0.05) contributes to the separation observed in the scores plot. The direction of the coefficient vector maps directly to the direction of the data points in the scores plot relative to the origin.

PC-CVA was performed to determine between-group multivariate differences in plasma metabolite profiles with respect to condition and genotype (Figure 3A). Overall, ∼65% of the total variance in the overall metabolomics dataset was explained by canonical variate (CV) 1, while CV2 explained ∼3% of the total variance. The PC-CVA scores plot showed significant differences in the multivariate mean between the rested and exercised conditions for both WT and DKI, as well as significant differences between WT and DKI plasma at rest, but no significant difference between WT and DKI following exercise. CV1 particularly demonstrates a more pronounced change in metabolic profile in DKI rested versus exercise, compared to WT rested versus exercised. The rested WT sample shows little change (sitting at the origin of the plot) relative to which the rested DKI sample are significantly different. When both genotypes are exercised their metabolic profiles change significantly but converge such that there was no significant difference between the exercised WT and DKI samples. The contribution of each metabolite (with 95% confidence intervals) to CV1 and CV2 are presented in Supplementary Figure S1 (significant metabolites indicated in red). Those metabolites observed to be significantly contributing to the model are summarized in Figure 3B. Here, the direction of each metabolite relative to the origin can be mapped to similar directions of sample groups in the scores plot (Figure 3A). As such, metabolite grouping along the x-axis are causal of differences associated with the exercise intervention (e.g., histidine to the left, reduced with exercise, and progesterone to the right, increased with exercise); whereas metabolites on the y-axis are more indicative of differences in rested genotype but no change in exercised genotype. The distribution of metabolites in Figure 2B reflect similar response clustering as the HCA results (Figure 2A). Cluster groups in Figures 2A, 3B are color-matched and the significant CV coefficients are labelled to the HCA plot.

## 4 Discussion

The metabolic pathways associated with the disruption of AMPK-glycogen interactions *in vivo*, despite clear phenotypic and physiological effects of the AMPK KI and DKI mutations (Hoffman et al., 2020; Janzen et al., 2021; Janzen et al., 2022), remain unknown. Furthermore, how an acute bout of exercise affects the plasma metabolome in widely used mouse models such as WT C57BL/6J mice, as well as the underlying metabolic pathways that contribute to these exercise-induced changes in plasma metabolite signatures has not been determined. In this study, we first reproduced the findings in AMPK DKI mice showing increased total body mass and fat mass (Janzen et al., 2021) and reduced maximal running speed (Janzen et al., 2022) relative to WT control mice (Figures 1B–E). We then performed metabolomics analysis on the plasma of WT and DKI mice both at rest and after acute exercise to investigate the metabolic mechanisms underlying increased adiposity and reduced exercise capacity in DKI mice. The metabolomics analyses revealed a significant mean metabolic difference between WT and DKI at rest but not after exercise, and a significant mean difference between rest and exercise in both genotypes. Cluster C metabolites (acylcarnitines, steroid hormones) increased significantly in both genotypes in association with exercise. In Cluster F (amino acids and derivatives), there was a significant interaction between genotype and exercise.

DKI mice evidenced a larger metabolome difference than the WT mice between rest and post-exercise conditions (Figure 3B). These differences support our working hypothesis that differences in substrate utilization and/or availability contribute to the overall phenotypic differences between DKI and WT mice. The PC-CVA CV1 scores for the rested DKI mice are further away from all exercised mice than the rested WT mice. This indicates that DKI mice may have a more “sedentary” resting metabolic phenotype compared to WT mice. This is supported by previous findings showing that DKI mice exhibit increased adiposity and total body mass, and reduced voluntary ambulatory activity versus WT mice (Janzen et al., 2021).

During exercise, cellular energy demand increases. While lipids are the primary fuel source at rest and during low intensity exercise (<50–60% VO_2_ max), carbohydrates utilization dominates at higher intensities (>70–75% VO_2_ max (van Loon et al., 2001; Hargreaves and Spriet, 2020). When exercise is prolonged or under conditions of low carbohydrate availability, amino acid contribution as fuel source is increased (Howarth et al., 2010). Steroid hormones such as the glucocorticoid corticosterone increase during times of physiological energy stress, such as exercise, and promote energy store mobilization by stimulating lipolysis, gluconeogenesis and protein breakdown (Girard and Garland, 2002). Exercise-induced lipolysis leads to increased circulating levels of free fatty acids and is also associated with increased acylcarnitines (Hargreaves and Spriet, 2020; Belhaj et al., 2021); this has also been shown in a recent investigation of rat plasma metabolomic responses to acute aerobic exercise (Overmyer et al., 2015). Both WT and DKI exhibited exercise-associated increases in steroid hormones and acylcarnitines (Cluster C). While we previously showed that DKI mice have decreased levels of whole-body lipid oxidation during fasting (Janzen et al., 2021) and submaximal exercise (Janzen et al., 2022), the present findings reveal no differences in lipid mobilization responses (both at rest and exercise) between the two genotypes. However, a more detailed lipid profile analysis may help establish potential differences in lipid utilization between genotypes.

Our previous findings also showed that DKI mice had increased depletion of skeletal muscle glycogen and increased reliance on carbohydrate oxidation during exercise relative to WT mice (Janzen et al., 2022). In the present study, a significant interaction (*p* = 3.53 $\times 10^{-4}$ ) was observed between genotype and exercise in amino acids (Cluster F), with DKI mice evidencing greater amino acid uptake and/or utilization in DKI versus WT mice in association with exercise (Figure 2B). Together, these observations may explain the reduced maximal exercise capacity in DKI versus WT mice (Figure 1B), as increased amino acid catabolism can help sustain the energy demands of exercise when preferred fuel sources decrease (Lewis et al., 2010; Stander et al., 2018), causing reduced exercise capacity. Specifically, decreased ketogenic (leucine and lysine) and glucogenic (histidine, arginine, proline, methionine) amino acids, and isoleucine (ketogenic and glucogenic) are generally reduced following exercise in blood and tissue samples from humans (Contrepois et al., 2020; Schranner et al., 2020) and rodents (Overmyer et al., 2015). Decreased amino acids may reflect increased ketogenesis and gluconeogenesis permitting continuous energy conversion when carbohydrate availability is reduced, as observed in DKI versus WT mice following exercise (Janzen et al., 2022). The precise mechanisms by which disrupting AMPK-glycogen interaction results in differential amino acid metabolism remain unclear and require further investigation.

The Cluster F metabolite pantothenic acid (vitamin B5) was significantly decreased in DKI versus WT plasma, and following exercise versus rest in both genotypes despite no interaction between genotype and exercise status. Pantothenic acid is a precursor for coenzyme A (CoASH), which is involved in numerous metabolic reactions, including energy metabolism and several steps in fatty acid oxidation (Garland et al., 1970; Leonardi and Jackowski, 2007; Watkins and Caballero, 2013). While the mechanisms underlying decreased plasma pantothenic acid levels in DKI versus WT mice remain unclear in the present study, this may involve potential differences in gut microbiome between genotypes, as pantothenic acid is produced by gut bacteria (Magnusdottir et al., 2015; Yoshii et al., 2019) and changes in the gut microbiome can impair pantothenic acid production (Gominak, 2016). Given the influence of gut microbiota on glucose metabolism and skeletal muscle function in mice (Nay et al., 2019), further research characterizing gut microbiome composition would help determine its potential contribution to the phenotypic differences observed in DKI versus WT mice including impaired maximal running speed [Figure 1B; (Janzen et al., 2022)], increased adiposity [Figure 1D; (Janzen et al., 2021)], glucose intolerance (Janzen et al., 2021) and changes in whole-body and skeletal muscle fuel utilization (Janzen et al., 2021; Janzen et al., 2022). Reduced pantothenic acid availability may also be associated with reduced free CoASH (Robishaw and Neely, 1985) and/or availability of subcellular pools of CoASH in skeletal muscle. Given the numerous critical roles of CoASH in energy metabolism, CoASH has been suggested as a potential limiting factor of substrate utilization and exercise capacity (Whitfield et al., 2021). The potential contribution of decreased skeletal muscle free CoASH to the decreased whole-body rates of fat oxidation and increased rates of carbohydrate oxidation observed in DKI versus WT mice (Janzen et al., 2021) remains unclear. Future research investigating CoASH species in skeletal muscle and its cytosolic and mitochondrial compartments is therefore warranted to determine their potential association with these phenotypic differences between DKI and WT mice.

A total of 17 metabolites were significantly different in association with exercise (Table 2). Although these were primarily metabolites that were expected to change with exercise (e.g., lipid species, amino acids and steroid hormones), it is worth noting two metabolites that have not been previously reported in association with exercise. N-acetyltryptophan is produced from tryptophan by the gut microbiota (Pavlova et al., 2017; Bihlmeyer et al., 2021) and exhibited a significant increase in association with exercise in both genotypes (Cluster C, Figure 2B). While the physiological consequences of these increases are not known, recent research supports the exchange of metabolites between the bloodstream and the guts (Tabone et al., 2021) and an exercise-induced bi-directional crosstalk (Mach et al., 2021). Glycerophospho-N-palmitoyl ethanolamine (GP-NPEA) is a glycerophospholipid membrane constituent with signal transduction roles *via* palmitoylethanolamide (Zhang et al., 2018). The mechanisms underlying changes in these plasma metabolites following exercise are unclear and further investigations are required to pinpoint their potential roles in exercise metabolism.

While this study characterized plasma metabolomic responses in DKI and WT plasma at rest and in response to exercise, some limitations need to be acknowledged. First, the metabolites reported in this study were limited to those that could be identified/annotated using an in-house metabolite library or based on MS/MS spectral patterns from external databases. Accordingly, the scope of our biological interpretations remains limited. Second, this study was powered based on the detection of differences in maximal running speed and may be insufficient to detect subtle effects in some metabolic pathways. Third, mice were randomly allocated to each group in the fed state at the start of the light cycle, meaning the timing of each mouse’s last food intake may have been different, therefore potentially contributing to variance in metabolite levels between mice at rest and/or following exercise at the time of sample collection. Fourth, to ensure sufficient blood volume required for metabolomic analyses could be collected *via* cardiac puncture, CO_2_ exposure prior to euthanasia was utilised. While this CO_2_ exposure could potentially impact plasma pH and metabolites, all mice were subjected to the same duration of CO_2_ exposure and we do not expect CO_2_ contributed to between-group differences. Finally, metabolomics studies analyzing biofluids such as plasma cannot provide comprehensive insight in terms of mechanistic regulation as, for many metabolites, little is known regarding the release and/or uptake regulation into tissues in response to a stimulus such as exercise. For example, the potential association of reduced plasma pantothenic acid levels in DKI mice with reduced skeletal muscle CoASH levels and/or rates of fat oxidation remains unclear, as we did not measure skeletal muscle metabolites in the present study. Therefore, complementary future analyses of metabolites in metabolically active tissues such as skeletal muscle will allow more comprehensive insights into how acute exercise and disruption of AMPK-glycogen binding affects tissue and circulating metabolomic signatures in mice.

## 5 Conclusion

This is the first study to map the plasma metabolomic responses to acute exercise in WT mice and simultaneously investigate the effects of disrupting AMPK-glycogen interactions on mouse plasma metabolome. Consistent with previous findings (Janzen et al., 2021; Janzen et al., 2022), we demonstrate that DKI mice have increased total body and fat mass, associated with reduced maximal running speed. A total of 83 mouse plasma metabolites were identified/annotated, of which 17 were significantly changed in association with acute exercise, including acylcarnitines, amino acids and steroid hormones. This study also identified/annotated previously unreported exercise-associated metabolites (e.g., GP-NPEA and N-acetyltryptophan). Metabolic differences associated with exercise were primarily explained by increased abundance of acylcarnitines and steroid hormones, and decreased abundance of amino acids. DKI mice displayed greater decrease in amino acids following exercise, potentially indicating increased utilization versus WT mice. DKI mice also displayed decreased pantothenic acid levels, which may indicate decreased levels of tissue pantothenic acid, a precursor of CoASH involved in multiple steps of energy metabolism. Whether the observed decrease in plasma pantothenic acid is associated with decreased CoASH in skeletal muscle is unknown, and further research is warranted to determine the potential contribution to differences in substrate utilization and exercise capacity observed in DKI versus WT mice. Future complementary investigation of the metabolome in metabolically active tissues such as skeletal muscle will provide further insights into the complex metabolite responses to acute exercise and phenotypic effects of disrupting AMPK-glycogen interactions on energy homeostasis and exercise capacity.

## Data Availability

The original contributions presented in the study are included in the article/Supplementary Material, further inquiries can be directed to the corresponding authors.
