# Supplementary material for: Metabolomics reveals mouse plasma metabolite responses to acute exercise and effects of disrupting AMPK-glycogen interactions
Source: Front Mol Biosci. 2022 Aug 24;9:957549. doi: 10.3389/fmolb.2022.957549 (PMC9449498; doi:10.3389/fmolb.2022.957549)
Supplement: Supplementary file 1 [file DataSheet1.zip › Frontiers_Belhaj et al. (2022) - Supplementary Material.pdf]

frontiers

Supplementary Material

Figure S1. Related to Figure 3. Loading plot showing metabolites that significantly contributed to CV1 and CV2. Data are represented as mean loading values within 95% CI. Metabolites that significantly contribute to CV1 and/or CV2 are represented by red-colored dots and CIs, while blue dots and CIs represent metabolites that do not significantly contribute their respective CV. Metabolites are sorted according to HCA clusters A to F (top-down). CI: confidence interval, CV: canonical variate, HCA: hierarchical cluster analysis.

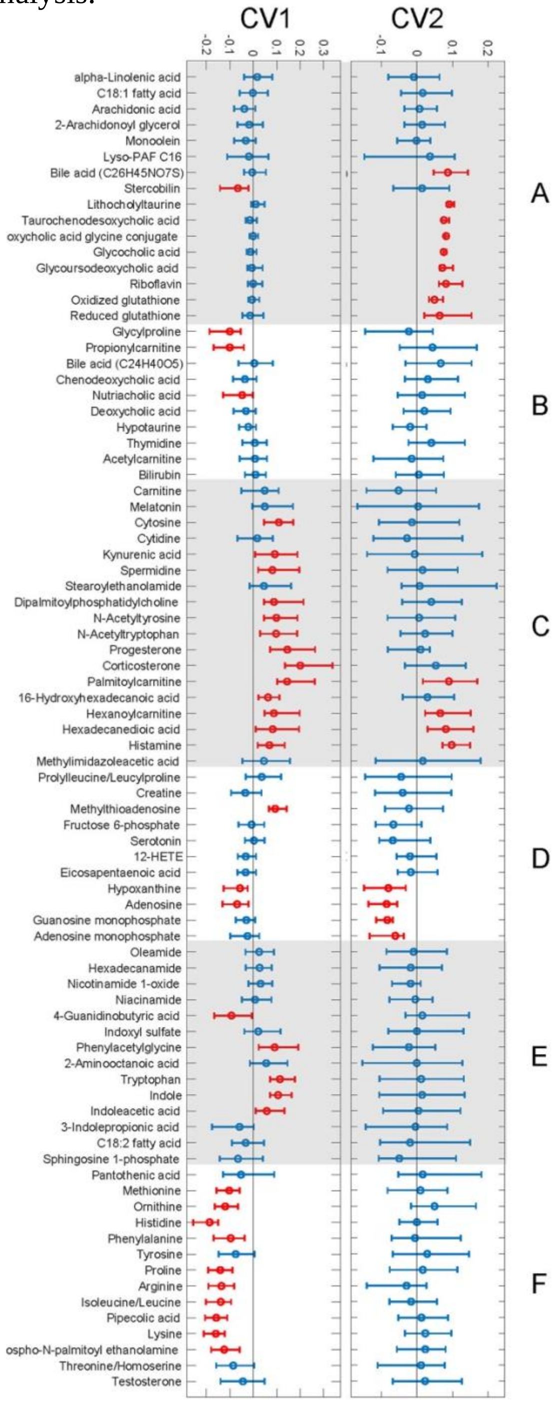

Table S1. Metabolite information

Table S2. Metabolomics Standard Initiative ID level

Table S3. Statistical Analysis by Group CV: canonical variate *: metabolites significantly contributing to CV1; #: metabolites significantly contributing to CV2. Significantly changed values and metabolites ($p<0.05$ and FDR<0.1) are shown in red text. Metabolites in magenta indicate significant changes for both genotype and condition.
